# COVID-19 severity is associated with population-level gut microbiome variations

**DOI:** 10.3389/fcimb.2022.963338

**Published:** 2022-08-23

**Authors:** Eva Lymberopoulos, Giorgia Isabella Gentili, Sanjay Budhdeo, Nikhil Sharma

**Affiliations:** ^1^The Sharma Lab, Department of Clinical and Movement Neurosciences, Queen Square Institute of Neurology, University College London, London, England; ^2^Centre for Doctoral Training in AI-London enabled Healthcare Systems, Institute of Health Informatics, University College London, London, England; ^3^National Hospital for Neurology and Neurosurgery, Queen Square, London, England; ^4^School of Biomedical Engineering & Imaging Sciences, Faculty of Life Sciences & Medicine, King’s College London, London, England

**Keywords:** gut microbiome, COVID-19, population health, topological data analysis, probiotics

## Abstract

The human gut microbiome interacts with many diseases, with recent small studies suggesting a link with COVID-19 severity. Exploring this association at the population-level may provide novel insights and help to explain differences in COVID-19 severity between countries. We explore whether there is an association between the gut microbiome of people within different countries and the severity of COVID-19, measured as hospitalisation rate. We use data from the large (n = 3,055) open-access gut microbiome repository curatedMetagenomicData, as well as demographic and country-level metadata. Twelve countries were placed into two groups (high/low) according to COVID-19 hospitalisation rate before December 2020 (ourworldindata.com). We use an unsupervised machine learning method, Topological Data Analysis (TDA). This method analyses both the local geometry and global topology of a high-dimensional dataset, making it particularly suitable for population-level microbiome data. We report an association of distinct baseline population-level gut microbiome signatures with COVID-19 severity. This was found both with a PERMANOVA, as well as with TDA. Specifically, it suggests an association of anti-inflammatory bacteria, including *Bifidobacteria* species and *Eubacterium rectale*, with lower severity, and pro-inflammatory bacteria such as *Prevotella copri* with higher severity. This study also reports a significant impact of country-level confounders, specifically of the proportion of over 70-year-olds in the population, GDP, and human development index. Further interventional studies should examine whether these relationships are causal, as well as considering the contribution of other variables such as genetics, lifestyle, policy, and healthcare system. The results of this study support the value of a population-level association design in microbiome research in general and for the microbiome-COVID-19 relationship, in particular. Finally, this research underscores the potential of TDA for microbiome studies, and in identifying novel associations.

## 1 Introduction

Despite the vast advances made in researching COVID-19 over the first two years of the pandemic, many of the mechanisms underpinning its severity and inter-individual variations in disease course are incompletely understood. This made it difficult to predict the likely severity within a given population and to plan patient flow at a regional and national level. Here, we explore the link between variations in the gut microbiome between countries and COVID severity. This is important as the gut microbiome is amenable to manipulation at the population level, potentially providing a novel intervention in future outbreaks and pandemics.

There are significant variations in the response to COVID-19. For example, while the disease predominantly affects the respiratory tract, it has been found to also cause a range of other symptoms, including non-specific (such as fever and headaches), neurological (such as anosmia and dysgeusia) or gastrointestinal (GI) symptoms (such as abdominal pain, vomiting, and diarrhoea) (Bostancıklıoğlu, 2020; Molina-Mora et al., 2022). Additionally, patients can experience rapid worsening associated with the so-called cytokine storm (Ragab et al., 2020) which is treated during a hospital stay (Ramiro et al., 2020; Cron et al., 2021). The factors and their combination that lead to such severe courses of COVID-19 are not understood yet. Several risk factors have been discovered, spanning multiple axes. One of these axes is socioeconomic factors. These include gender, ethnicity, BMI, household income, and especially the intersection of such vulnerabilities (Booth et al., 2021; Chen and Krieger, 2021; Pijls et al., 2021). For example, people of minority ethnic status in the most deprived neighbourhoods have the worst prognosis when infected and are most likely to get infected in the first place (Berkowitz et al., 2020). The second axis relates to weakness of the immune system, for example in the elderly, chronically ill, disabled, and immunocompromised (Mohammed et al., 2020; Baek et al., 2021; Booth et al., 2021). The microbiome is a further variable that appears to affect the severity of a COVID-19 infection but has received less attention so far (Segal et al., 2020).

The gut microbiome is a collection of an estimated 38 trillion microbes (Sender et al., 2016) colonising mostly the intestines, which have vast effects on host health. By metabolising food, they produce many key metabolites for physiological functioning across several organs and body systems. There is a particular interaction of the microbiome with immune pathways (Thaiss et al., 2016; Levy et al., 2017). As such, it has become the focus of research on inflammatory diseases, including chronic infections, autoimmune diseases, metabolic, and even psychiatric and neurodegenerative diseases (Wang et al., 2017). The microbiome has a key translational advantage over other research targets in these diseases as it is easily measured and modifiable by diet.

The gut microbiome is an intriguing candidate for a unifying risk factor, or even mediator, of COVID-19. When considering the epidemiological triad, it is part of both host factors, and the effect of environmental factors on the host.

For one, there is a complex relationship with social risk factors. For example, the microbiome varies by ethnicity (Gupta et al., 2017; Deschasaux et al., 2018), socioeconomic status (Bowyer et al., 2019), and BMI (Falony et al., 2016). It also changes with age (Yatsunenko et al., 2012; Zapata and Quagliarello, 2015; Badal et al., 2020). Several medical conditions which increase the risk of COVID-19 are also associated with changes to gut microbiome composition, leading to the hypothesis that the microbiome is a mediator for the disease-risk association (Kim, 2021). Another piece of evidence of the involvement of the gut microbiome is the reported GI symptomatology in COVID-19 (Villapol, 2020), likely through gut epithelial cells which express ACE-2 receptors, a major entryway for SARS-CoV-2 (Wang et al., 2020; Guney and Akar, 2021). Further, SARS-CoV-2 is found in the stool of COVID-19 patients (Wu et al., 2020). The gut microbiome is a major modulator of the innate immune system, with metabolites of the gut affecting intestinal barrier integrity, as well as the immune system itself (Jiao et al., 2020). These metabolites have been found to directly up- or downregulate cytokines, which lends some bacteria the labels anti-inflammatory or pro-inflammatory (Wang et al., 2020). In physiological homeostasis, these bacteria are thought to regulate each other through competition and quorum sensing, resulting in normal functioning. However, in an imbalanced state, these control mechanisms are reduced, and pro-inflammatory bacteria and pathogens can increase exponentially, a phenomenon called ‘blooming’. An altered microbiome is also often associated with a reduction in the diversity of bacteria in the gut (Belizário and Faintuch, 2018), though this is likely an oversimplification.

Immune-modulatory effects of the gut microbiome have been found to play a role in small studies of COVID-19 patients. A study from the USA compared patients of various levels of severity and controls (n = 48), reporting a significant decrease in anti-inflammatory and increase of pro-inflammatory species in symptomatic patients. Specifically, they found a reduction of *Bifidobacterium* and *Faecalibacterium* in patients that was inversely predictive of disease severity, as well as an increase in *Bacteroides* which was also predictive of severity (Hazan et al., 2022). In hospitalised patients, a similar decrease in commensal symbionts and an increase in opportunistic pathogens (compared to healthy controls) was reported. Of note, it did not resolve when the infection cleared (Zuo et al., 2021). Additionally, these patients show an increase in gastrointestinal symptoms (Bozkurt and Bilen, 2021a). While such reports show that the microbiome differs in more severely ill patients, they cannot explain the causality of this association. Does COVID-19 cause microbiome changes, or does variation in the microbiome at baseline influence severity?

Some evidence suggests that gut bacteria have a direct influence on COVID-19 severity through regulating cytokines. This is a well-known function of the gut microbiome, mostly by regulating the T-helper 17 (Th17) and T-regulatory (Treg) cell balance Omenetti and Pizarro, 2015. For example, a retrospective study of a probiotic booster administered to hospitalised patients reduced the number of inpatient days and the mortality rate, and improved radiological findings Bozkurt and Bilen, 2021b. This study also observed reduced levels of Interleukin-6 (IL6), a key pro-inflammatory cytokine of the innate immune response that has been implicated in increased severity of COVID-19 Coomes and Haghbayan, 2020 and the COVID-19 associated cytokine storm Ragab et al., 2020. Such evidence suggests that the microbiome could in fact have a causal relationship with COVID-19 severity through regulating the immune response.

Small sample size is a significant limitation to the current literature. Studies have typically been conducted at the individual level with small sample sizes (n<30). This introduces major issues as there is considerable inter-subject variability driven in part by the huge scale of the microbiome, as well as its complex nonlinear relationships. This is exacerbated by microbiome analyses that focus on differences in abundance between groups. Additionally, these studies are underpowered for detecting key confounding factors such as demographic, socioeconomic, or medical factors.

Examining the gut microbiome at a population level may provide an elegant solution to address the limitations of small populations and could provide a target for public health interventions. For one, this approach allows sample sizes of several thousand participants. Additionally, by shifting the focus from individuals to populations, we can consider how the microbiome might affect the differences in COVID-19 severity that were observed across countries. Indeed, it is known that the microbiome of the population varies between countries. This is influenced by differences in local water and soil microbiomes, diet and other lifestyle factors, antibiotic prescription patterns, hygiene, and pollution (Karlsson et al., 2014; Gupta et al., 2019; Lymberopoulos et al., 2021). By considering the link between the gut microbiome and COVID-19 severity, it is possible that the microbiome of a population influences the level of COVID-19 severity experienced in that population. In fact, the impact of the COVID-19 pandemic varied strongly between countries. While the rate of infection was influenced by governmental response and strictness of public health measures such as lockdowns (Huang et al., 2021; Arnold et al., 2022), as well as other population metrics (Sorci et al., 2020), there remains a difference in experienced severity across countries with similar rates of infection.

While there is now evidence to suggest a link between the gut microbiome and COVID-19 severity, current evidence is limited by the size and design of individual-level studies which can be addressed with a population-based approach. We aim to explore whether country-level differences in the microbiome before the pandemic can explain a degree of the country-level differences in COVID-19 severity, as measured by hospitalisation rate. In addition to standard microbiome analysis, we employ an unsupervised machine learning tool - Topological Data Analysis - which is particularly appropriate for large microbiome datasets and can detect even subtle non-linear and mixed effects (Liao et al., 2019).

We hypothesise that there will be significant differences in microbiome composition between countries based on COVID-19 severity grouping that will be evident with standard analysis as well as TDA. We expect to find specific microbiome signatures to be associated with such groups of low and high severity. Specifically, we hypothesise that TDA will find more complex associations above and beyond those results from standard analysis. Finally, we expect to be able to discern the effects of relevant confounders.

## 2 Methods

### 2.1 Data

#### 2.1.1 Microbiome data

This study used an open microbiome data source, curatedMetagenomicData (cMD) accessed through BioConductor (Pasolli et al., 2017). This dataset used 16S rRNA amplicon sequencing. Because of its highly conserved nature, this technique now represents the standard application to the investigation of the microbiome (Gupta et al., 2019).

For this study, healthy participants over the age of 2 years old were included. In the first 2 years of life, the gut microbiome undergoes drastic shaping to its structure and functionality, particularly between 6 months and 2 years when solid foods are introduced, resulting in the final maturation stages of the microbiome (Robertson et al., 2019). Considering this, we excluded participants under 2 years old as their microbiome is still in the maturation phases and may therefore report misleading conclusions. Children over the age of 2 were not excluded as their microbiome is already more stable. Additionally, they are counted as part of hospitalisation data, and we matched the population of each dataset accordingly. The curat dataset comprised both age data and age category, which included newborns, school-age, children, and adults. In the case where subjects had missing data for both age and age category, they were excluded. The age group comprising newborns were fully excluded, as well as participants under the age of two. For ease of analysis, all participants’ age group was collected as metadata. Additionally, sample-level demographic data of gender and BMI were used.

We further excluded samples from countries with less than 100 samples, to ensure a level of representativeness of the microbiome data.

This filtering yielded 3,055 healthy gut microbiome samples from 12 countries. These were Canada, Denmark, Estonia, Finland, France, Great Britain, Israel, Italy, Netherlands, Spain, Sweden, United States of America.

#### 2.1.2 COVID-19 severity data

For each of these countries, COVID-19 severity data was obtained from Our World in Data (https://github.com/owid/covid-19-data), using hospitalisations as the preferred metric representing the number of COVID-19 patients in hospital on a given day. We chose severity to be operationalised by hospitalisations as it is the measure most likely to reflect the hypothesised immune-modulating effects of the microbiome, as opposed to factors relating to public policy or the healthcare system. These would have more influence on metrics of cases, test-positivity-rate, ICU beds, or fatality rates.

We restricted the data to comprise the hospitalisations before the initiation of the vaccination programme (before December 2020), to avoid potential confounding effects of vaccine roll-out. For each country, the number of absolute hospitalisations was summed over the whole period and then calculated as hospitalisations per 100,000 population.

**Figure 1:**
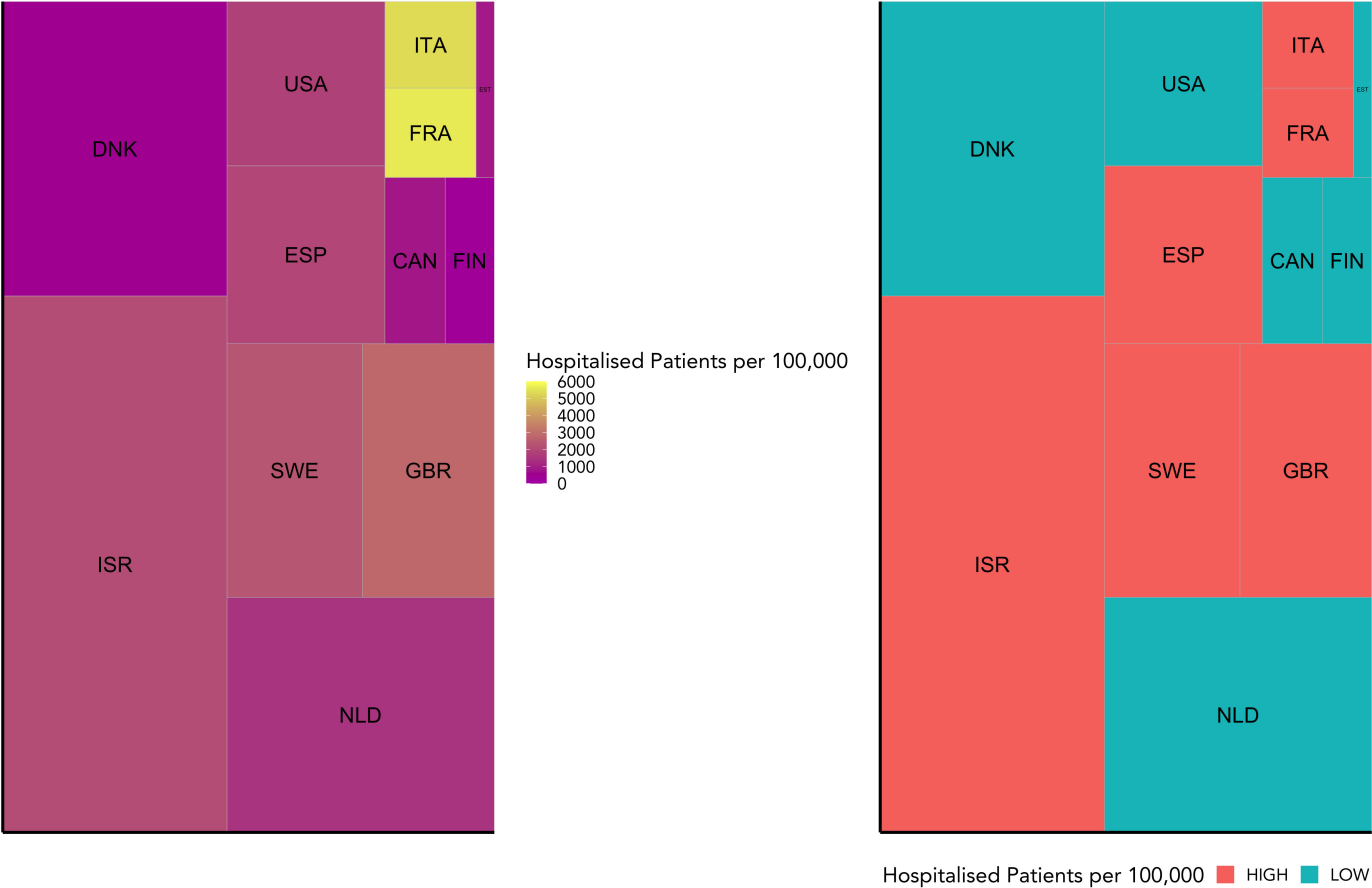
This figure visualises the weighted grouping of countries into COVID-19 severity groups. It shows the hospitalisation rate for each country included in this study relative to the number of microbiome samples available for that country. While the hospitalisation rate is indicated by colour, the sample size is represented by the area of each tile. On the left is the raw data, and on the right the grouping into high or low COVID-19 severity groups (high in red, low in blue) based on a weighted mean into even groups.

Using a weighted average, included countries were grouped into ‘high’ and ‘low’ COVID-19 severity groups. These include Canada, Denmark, Estonia, Finland, the Netherlands, and the USA in the high severity group, and France, Great Britain, Italy, Israel, Spain, and Sweden in the low severity group. This grouping is reflective of the data distribution as indicated in **Figure 1**.

Finally, country-level confounding variables were collected that could present relevant confounders in the association between the microbiome and COVID-19 severity. The first of these is the proportion of over 70-year-olds in the population, as the older population is more at risk of severe COVID-19 (Damayanthi et al., 2021). The second includes two measures of wealth and industrialisation level: the Gross Domestic Product (GDP) and the Human Development Index (HDI). These three measures were chosen as there is previous evidence that they are associated with COVID-19 fatality differences between countries which suggests potential relevance to COVID-19 severity in general (Sorci et al., 2020). Additionally, both age and wealth influence the microbiome (Bowyer et al., 2019; Badal et al., 2020), meaning there is potential for influence on the relationship between the microbiome and COVID-19, as well.

### 2.2 Analysis

Standard microbiome analysis was performed in R version 4.1.2 (2021-11-01) using the *curatedMetagenomicData* (3.2.3), *phyloseq* (1.38.0), and *microbiome* (1.16.0) packages. Unsupervised Machine Learning with TDA was performed in *Python 3.1.4* in a Jupyter Notebook environment, using mainly the *pandas* (1.2.4), *NumPy* (1.19.5), and *tmap* (1.0) *libraries and seaborne* (0.11.1) and *matplotlib* (3.3.4) for visualisations. All code is available on GitHub (*via* thesharmalab-team/microbiome_covid).

#### 2.2.1 Standard microbiome analysis

The composition of the gut microbiome by phylum was investigated using bar plots to observe differences in the overall relative abundance of phyla between the COVID-19 groups.

Alpha diversity compromises a mathematical measure of diversity within the microbiome population. This used the Shannon index to account for both species abundance and evenness (p<0.05 considered significant). This metric was compared within all individual included countries and by Covid-19 grouped countries.

Beta diversity analysis was run to measure the difference in microbiome composition between the high and low severity groups. This was analysed with the Bray-Curtis dissimilarity matrix and visualised with Principal Coordinate Analysis (PCoA).

A permutational multivariate analysis of variance (PERMANOVA) was adopted to identify the top genus-level taxa which significantly differ between the COVID-19 groups. This further allows for the overall significance testing between the microbiome composition and the COVID-19 groups (permutations = 999).

#### 2.2.2 Tmap

Topological Data Analysis (TDA) is an unsupervised machine learning tool based on algebraic topology and differential geometry (Carlsson, 2009). This means that it analyses the topological structure, or shape, of high-dimensional data without sacrificing complexity. TDA has many attributes that make it attractive for microbiome research, particularly its robustness to noise and sensitivity to signal. This enables it to detect even small and non-linear effects, as demonstrated in a previous study which showed that it outperformed standard microbiome tools (Liao et al., 2019). That study combined the TDA algorithm Mapper (Singh et al., 2007) with a modified version of the Spatial Analysis of Functional Enrichment algorithm (SAFE) (Baryshnikova, 2016) into a single library, *tmap*, for integrated stratification and association analysis of population microbiome data. This library is also used in this study.

The Mapper algorithm is a clustering method based on simplicial complexes that represent high-dimensional data, such as the microbiome data of this study, as a network graph. The input is a point cloud, with each microbiome sample constituting one data point. Through filtering, covering, and clustering in the high-dimensional space, data points are converted into nodes and edges. Nodes represent groups of samples with similar microbiomes which are close in the high-dimensional space, while edges represent overlap between these groups.

The SAFE algorithm, as used here, maps variables such as host metadata or bacterial taxa, onto the microbiome network as node attributes and generates subnetwork scores. These so-called SAFE scores exist for each node but can be transformed to a network-wide score called SAFE enriched score which enables comparison between variables, as well as co-enrichment in which relationships between variables are analysed.

The procedure used to implement tmap in this study is as follows. The first step is the optimisation of the cover ratio, which includes hand-tuning the parameters of overlap and resolution, as well as the minimum number of samples required to form a node. The cover ratio determines how sparse versus dense the graph network representation of the microbiome data will be. Based on these, the final Mapper network graph is calculated. Here, hand-tuning resulted in using a minimum number of samples of 7, resolution of 100 and overlap of 1.1. Then, all microbiome taxa and metadata variables are enriched using the SAFE algorithm. This is then used for metadata stratification, meaning the comparison of enrichment for groups. The final step is co-enrichment analysis, in which pairwise co-enrichment is calculated and a strict 0.5th percentile significance cut-off applied.

## 3 Results

### 3.1 COVID-19 severity

**Table 1** shows the severity of COVID-19 by total hospitalisations and hospitalisation per 100,000 before the commencing of the vaccination programme.

**Table 1:** Displayed in the table are the country-level variables for the 12 countries included in the analysis.

Country-level data					
Location	Proportion over 70 years old	GDP	HDI	Hospitalisations per 100,000	Frequency
Canada	10.80	44,017.59	0.93	803.68	75
Denmark	12.33	46,682.51	0.94	517.23	494
Spain	13.80	34,272.36	0.90	1,968.95	210
Estonia	13.49	29,481.25	0.89	868.86	24
Finland	13.26	40,585.72	0.94	258.09	61
France	13.08	38,605.67	0.90	5,621.29	61
Great Britain	12.53	39,753.24	0.93	2,830.04	250
Israel	7.36	33,132.32	0.92	2,086.07	900
Italy	16.24	35,220.08	0.89	5,364.83	59
Netherlands	11.88	48,472.54	0.94	1,401.75	470
Sweden	13.43	46,949.28	0.95	2,366.49	257
USA	9.73	54,225.45	0.93	1,816.21	194

This includes hospitalisation per 100,000 and the number of microbiome samples (Frequency) which are the basis of the grouping into low and high severity. Confounding factors are also included for each country: proportion over 70, GDP and HDI. These were obtained from the COVID-19 Dataset from Our World in Data.

Based on the data reported in **Table 1**, we allocated the 12 countries into two groups: high and low Covid-19 hospitalisation per 100,000. The separating limit used for the Covid-19 groups is 1842.75 hospitalisations per 100,000. As displayed in **Figure 1**, the low severity group includes Canada, Denmark, Estonia, Finland, the Netherlands, and the United States of America. The high severity group includes Spain, France, Great Britain, Israel, Italy, and Sweden. While the tiles are coloured according to the observed variables, the area of each tile represents the microbiome sample size for each country.

Presented in **Table 2** are the demographic factors for the cMD dataset (Gender, Age Group, and BMI) observed for the high and low Covid-19 grouping.

**Table 2:** The demographics table reported shows the number of samples for each variable by COVID-19 severity group.

Demographic Data by COVID-19 Severity Groups.			
Variable	Low Severity Group	High Severity group	Total
Gender			
Female	528	487	1015
Male	476	89	565
Unknown	314	1161	1475
Age Group			
Adult	1130	1546	2676
Child	88	92	180
Schoolage	66	3	69
Senior	34	96	130
BMI			
Lean (BMI 18.5+ to 25)	508	209	717
Overweight (BMI 25+ to 30)	230	114	344
Obese (BMI 30+ to 35)	146	43	189
Morbid obese (BMI 35+ to 40)	9	2	11
Severe Obese (BMI 40+ to 45)	25	11	36
Super Obese (BMI 45+)	16	11	27
Unknown	384	1347	1731

These are gender, age group, and BMI. These values were obtained from the cMD Dataset.

### 3.2 Standard microbiome analysis

**Figure 2:**
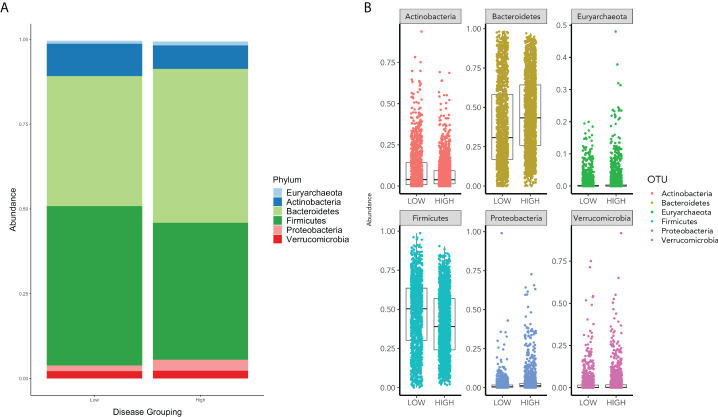
**(A)** displays the relative phyla abundance for the Low and High COVID-19 groups. **(B)** displays the relative abundance for each phylum for High and Low High COVID-19 groups.

In assessing the relative abundances of phyla, an increase in Bacteroidetes and Proteobacteria and a decrease in Firmicutes and Actinobacteria is shown in the high group in comparison to the low group (**Figure 2**).

**Figure 3:**
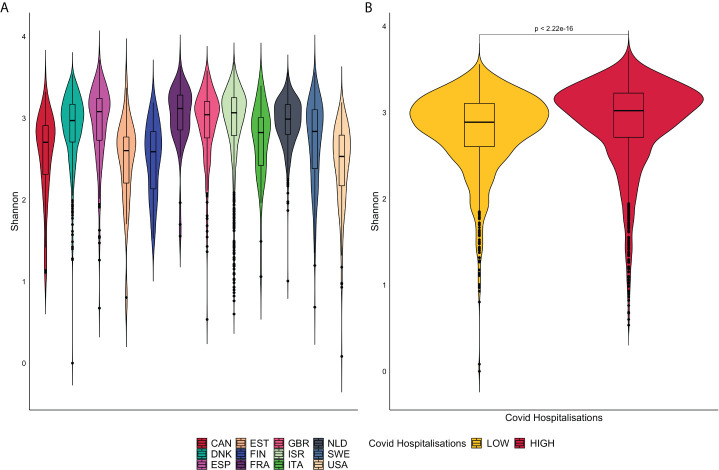
**(A)** is a representation of violin plots based on the Shannon alpha diversity index for each country. **(B)** displays violin plots based on Low and High COVID-19 groups with corresponding p-values.

The alpha diversity represented by violin plots in **Figure 3**, reveals significant differences, *via* the Wilcoxon Sign test (p<0.05), in microbiome diversity and distribution between the high and low groups. France presents with the greatest bacterial diversity and the lowest is associated with the United States of America.

**Figure 4:**
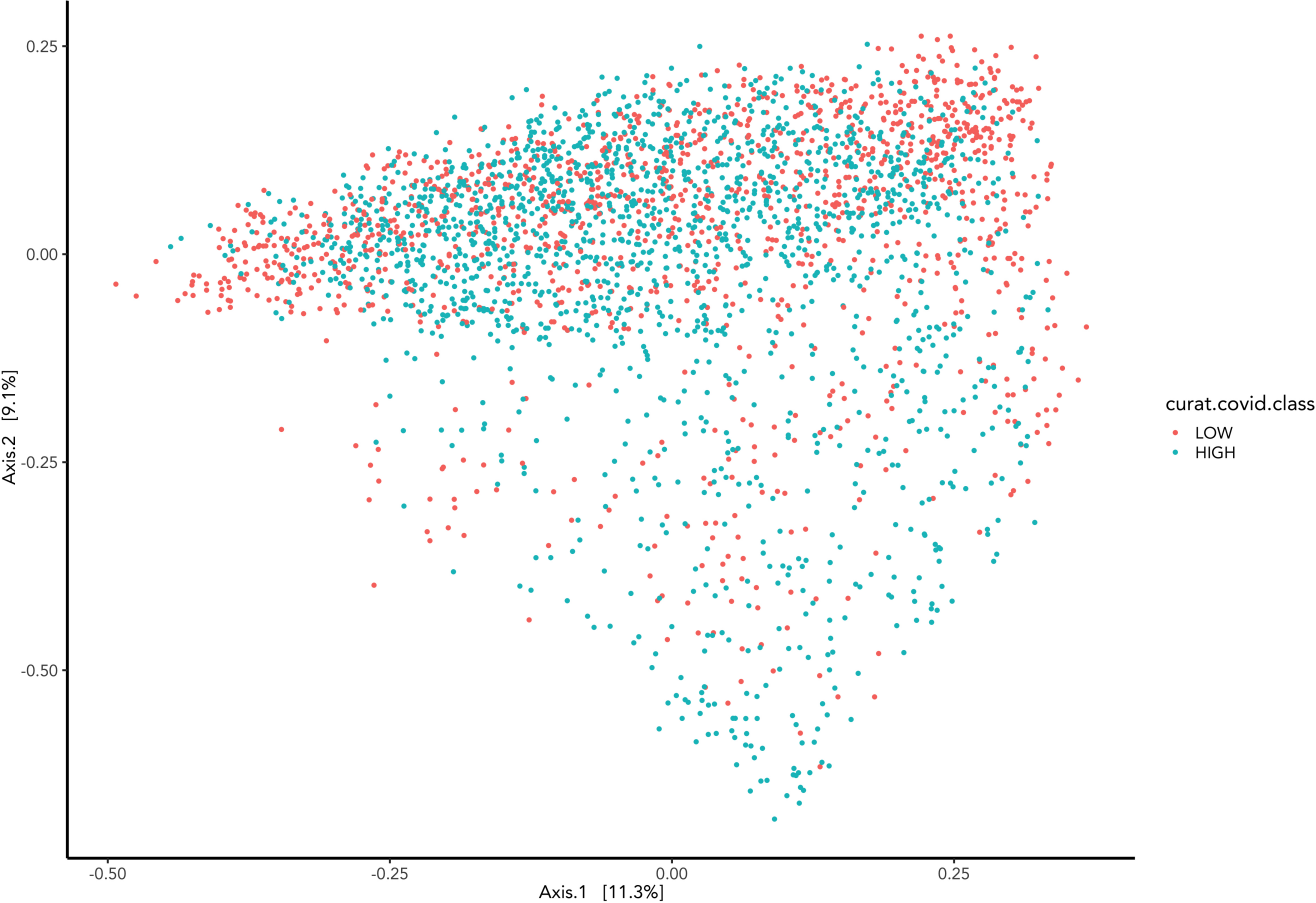
Represents the Bray-Curtis dissimilarity distance metric applied to PCoA graph representing the difference between samples grouped by COVID-19 hospitalisations.

Beta diversity visually presents microbiome composition variations and differences between Covid-19 groups (**Figure 4**). Samples are represented as points on the graph, their closeness implies their similarity in sequence composition. The clustering of the high and low groups appears to occupy the same region.

**Figure 5:**
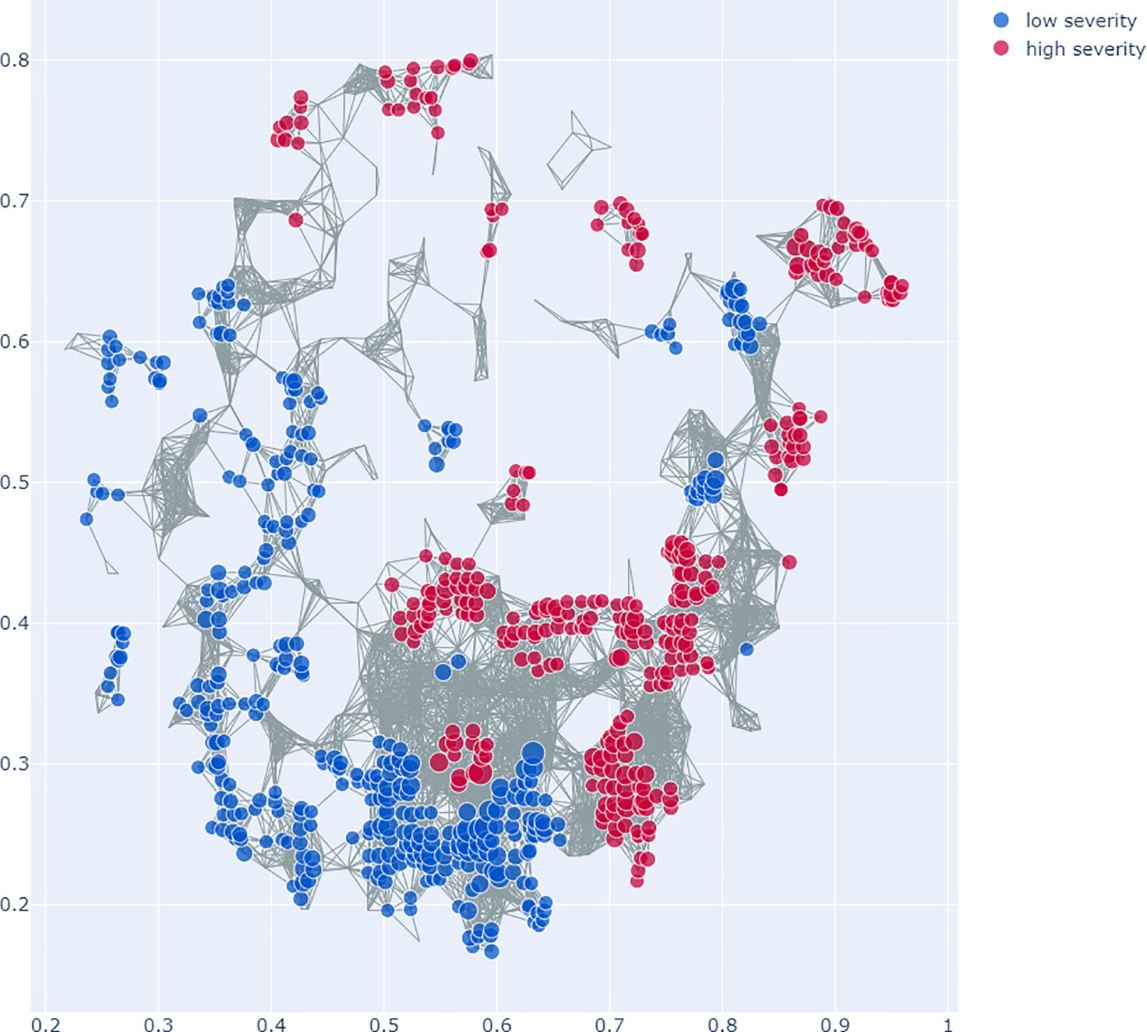
Top genus-level bacteria separating low and high COVID-19 groups. The bars towards the right represent higher abundance in the low COVID-19 group whilst the bars towards the left indicate higher abundance in the high COVID-19 groups. The x-axis in this figure displays the logarithmic change of bacteria which differed significantly.

Extending from the overall differences in phyla abundances between the high and low groups (**Figure 2**), differences in top genus-level taxa were explored and observed in **Figure 5**. A noticeable higher proportion of *Eubacterium rectale* dominates the low group contrarily to the *Prevotella copri* in the high group. Overall multiple species of the *Ruminococcus* genus prevail in the low group. Both groups include the genus *Bacteroides* although distinct species are associated with them. The second most abundant genus-level taxa associated with the high and low group are *Escherichia coli* and *Bifidobacterium adolescentis,* respectively.

### 3.3 Topological data analysis

The analysis with TDA Mapper yields a graph network of the microbiome data that contains 1,323 nodes and 11,258 edges, with 1,125 (63.18%) samples lost.

**Figure 6:**
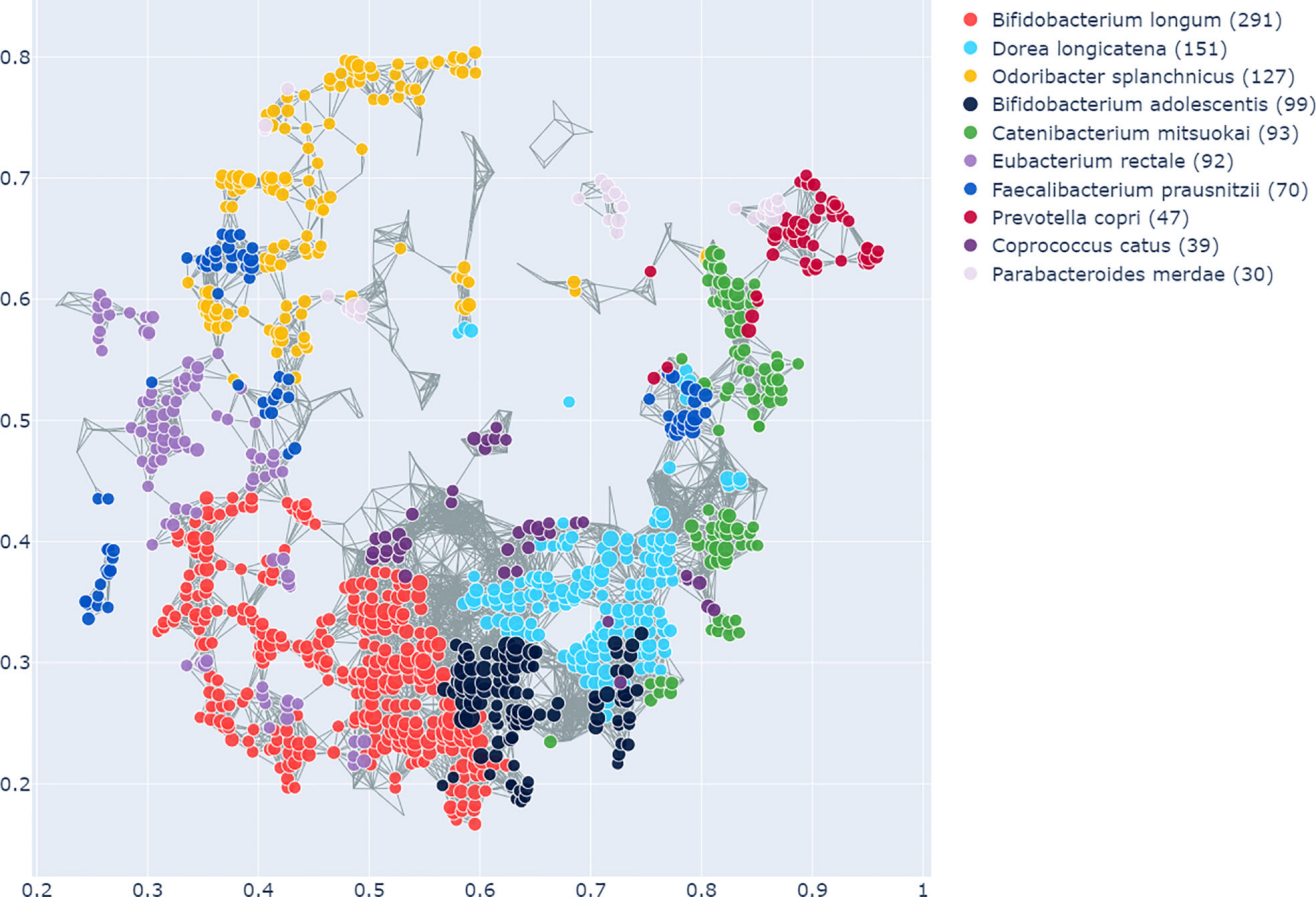
Topological Data Analysis (TDA) with Mapper: This figure shows the network graph made from the microbiome data. Each node represents a group of samples with similar microbiome profiles, each edge represents overlap in the groups. The colours represent the COVID-19 severity groups, enriched with the SAFE algorithm on the network. Specifically, which of the groups has the higher SAFE score at a given node. The figure shows that there is clear clustering of the groups across the network, with the low severity group mostly enriched across the lower left of the network graph. The high severity group is enriched in the right and top of the network graph. This suggests different microbiome profiles associated with the groups.

SAFE enrichment of the COVID-19 severity groups on the Mapper graph shows clustering of the groups in distinct areas of the network: while the low severity group is enriched in the lower left part of the network, the high severity group is enriched in several clusters in the right and top parts of the network (see **Figure 6**). This suggests that different microbiome profiles are associated with low and high severity.

**Figure 7:**
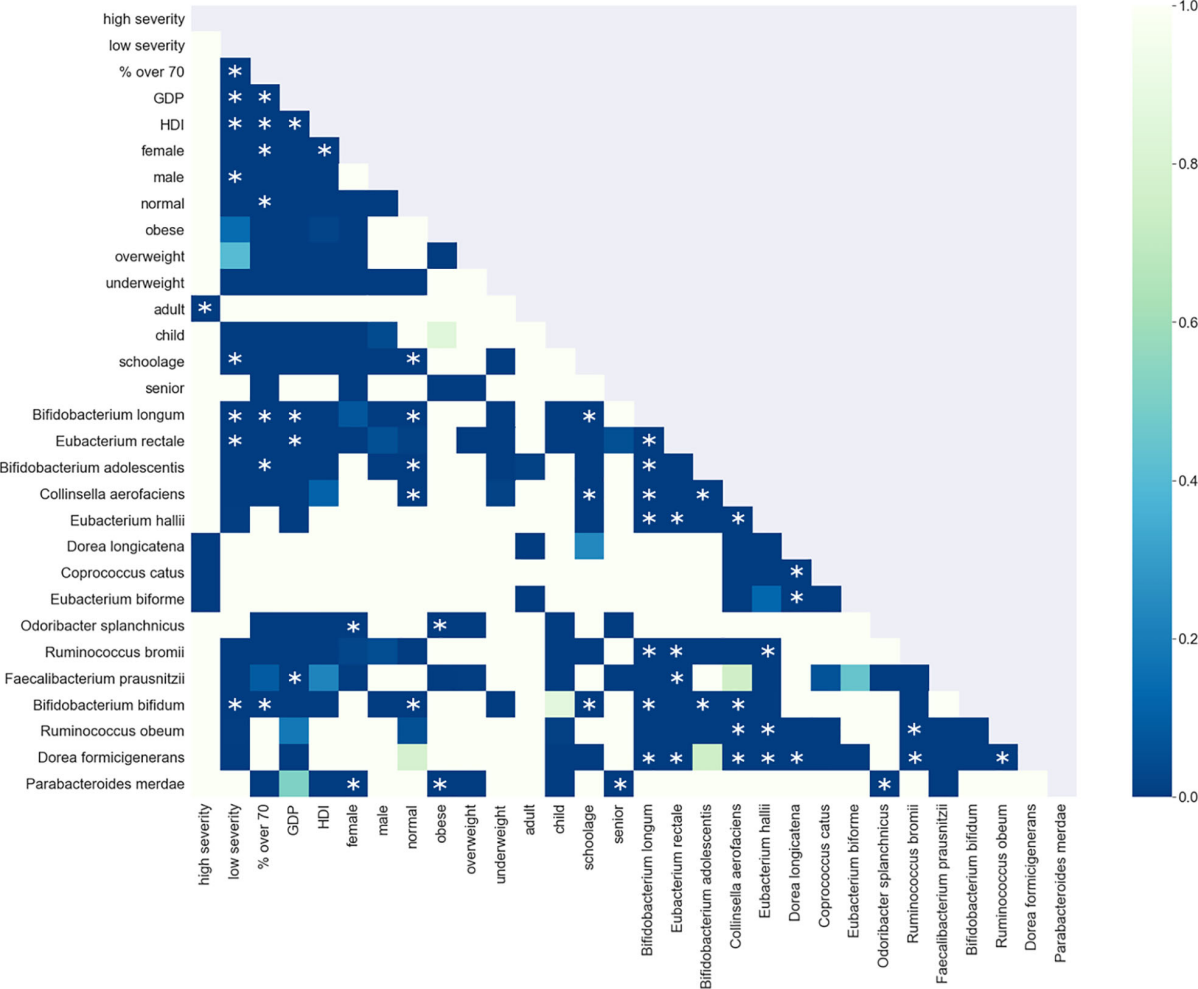
As in **Figure 6**, this shows the graph network constructed from the microbiome data with the TDA Mapper algorithm. This graph shows the bacterial taxa with the highest number of enriched nodes and the nodes in which a given taxon has the highest SAFE score are coloured accordingly.

**Figure 8:**
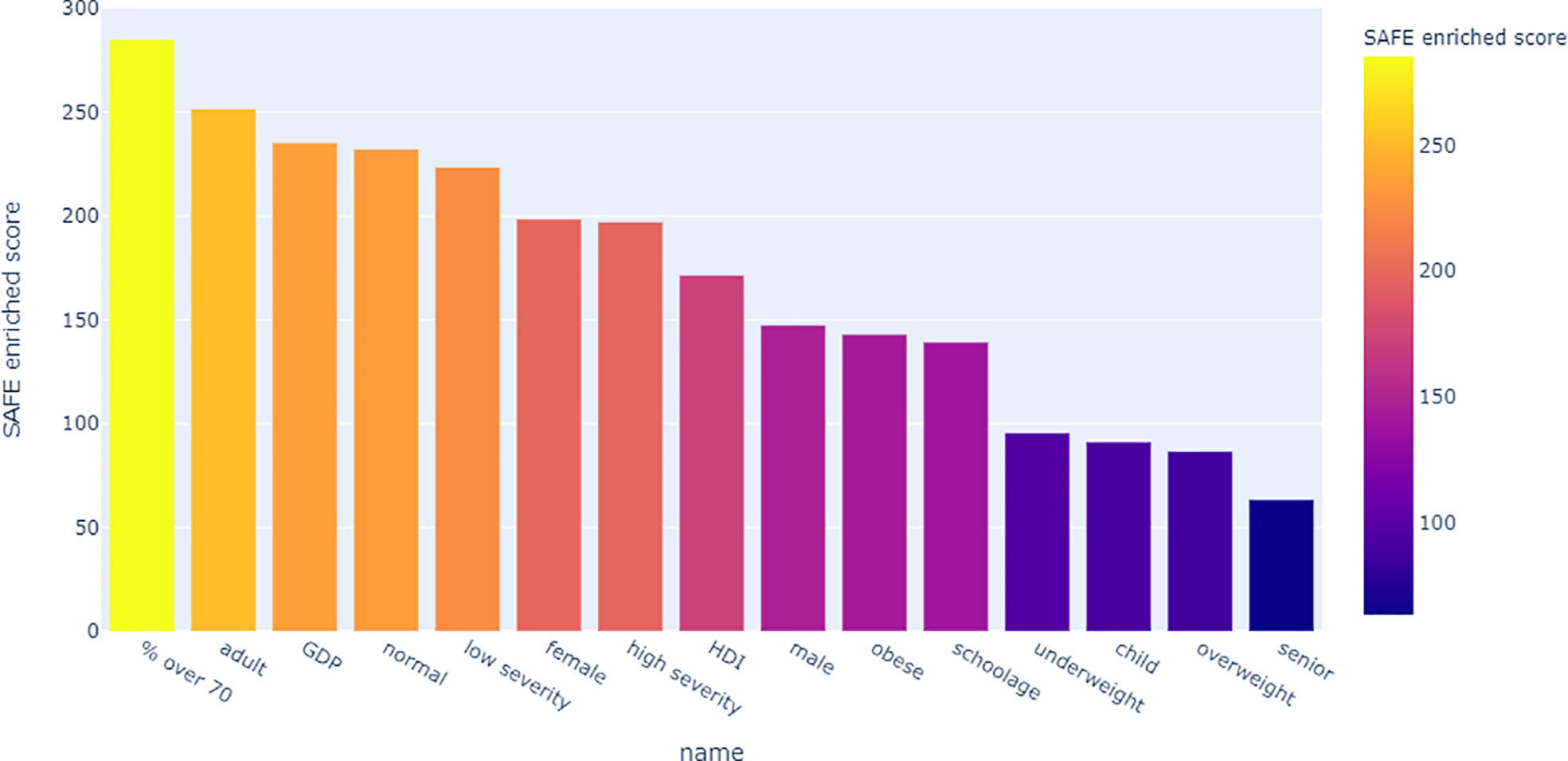
Shown is the association matrix of pairwise co-enrichment of all metadata and the 15 top taxa. The top taxa are a combination of the 10 taxa with the highest SAFE enriched scores (network-wide) and the 10 taxa with the highest number of enriched nodes. The colours represent the p-value, asterisks indicate significance at the 0.5th percentile of all pairwise associations (not all represented in the matrix). Of note, the low severity group is associated with the taxa *Eubacterium rectale, Bifidobacterium longum* and *Bifidobacterium bifidum*, and the population-level confounders GDP, HDI, and the proportion of over 70s in the population. These bacteria are also associated with each other, the same holds true for the confounders. Additionally, the bacteria and confounders are associated with each other and other anti-inflammatory bacteria such as *Bifidobacterium adolescentis* or *Faecalibacterium prausnitzii*.

The enrichment graph comparing the bacterial taxa with the most enriched nodes across the network (**Figure 7**) also shows distinct clusters of enrichment. Most of these bacteria are highly common in the human gut. The genus with the highest number of enriched nodes is *Bifidobacterium longum* which also has the highest SAFE enriched score. It is enriched in a large cluster in the bottom left of the network, as is for example *Eubacterium rectale* (second highest SAFE enriched score). *Dorea longicatena* on the other hand, the taxon with the second most enriched nodes and fifth highest SAFE score, is enriched in a cluster on the bottom right. While this graph visually suggests co-enrichment between the severity groups and some taxa, this can only be shown with pairwise co-enrichment analysis. The results of this are presented in the association matrix in **Figure 8**, with asterisks indicating statistically significant associations. While the high severity group only significantly co-enriched with the adult age category, the low severity group has several associations. It is associated firstly with three of the most highly enriched taxa, namely *Bifidobacteria longum* and *bifidum*, and *Eubacterium rectal*e. Further, it is associated with many metadata of interest, including the individual-level variables male gender and the schoolchildren age category, as well as the population-level variables proportion of over 70-year-olds in the population, GDP, and HDI. Of the taxa associated with the low group, the *Bifidobacteria* are associated with normal BMI, and all three taxa co-enrich with each other and taxa such as *Eubacterium hallii*, *Bifidobacteria bifidum* and *adolescentis*, and *Ruminococcus bromii*. The three population-level covariates all significantly co-enrich with each other. Proportion over 70 years old and GDP are also each associated with the key taxon *Bifidobacterium longum* and other anti-inflammatory bacteria. Further, the co-enrichment analysis shows clusters of association between several anti-inflammatory bacteria, and between pro-inflammatory bacteria. One interesting association is between *Collinsella aerofaciens* and *Bifidobacteria longum* and *bifidum*, which, as mentioned above, are both associated with the low severity group.

**Figure 9:**
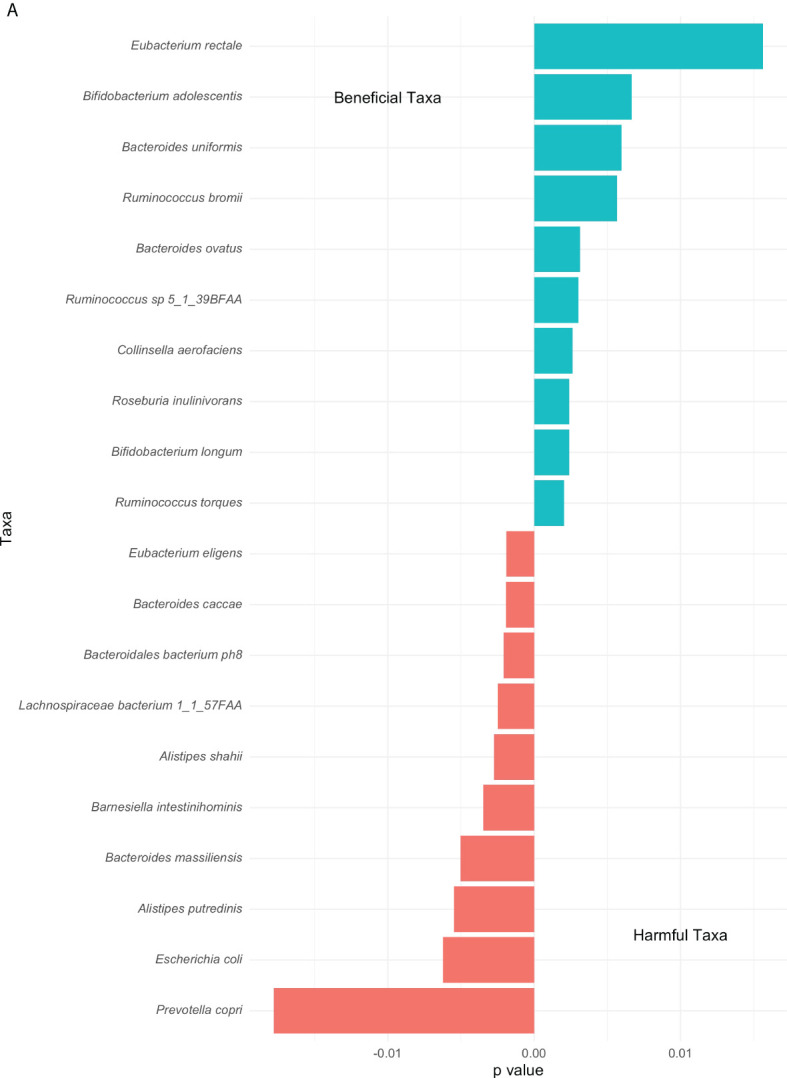
Bar chart of the network-wide SAFE enriched score of all metadata. This score is an indicator of effect size, allowing comparison across variables.

Looking at the SAFE enriched scores of the metadata, it shows that both country-level metadata, namely proportion over 70 and GDP, as well as individual-level metadata, namely adult age and normal BMI were more highly enriched than the covid severity grouping (**Figure 9**).

## 4 Discussion

This study is the first to apply a population-based indirect association approach to the association between the gut microbiome and COVID-19 severity, supported by the use of unsupervised machine learning with Topological Data Analysis. The study successfully elucidated significant differences between countries with low and high COVID-19 severity. We have detected microbiome signatures at the genus level which dominate the severity groups, as well as the influence of various confounding factors influencing this relationship. Microbiome composition varies significantly with geographical location, which is associated with a diverse range of diseases (Sun et al., 2020; Chen et al., 2021; Romano et al., 2021). Overall, our results support the complex association between the gut microbiome and COVID-19 severity.

This study comprises a large sample size of 3,055 gut microbiome samples which is vastly greater in comparison to that used by similar studies conducted at the individual level. Another major strength lies in the combination of two analysis approaches, both conventional and machine learning. This helps to strengthen results and conclusions, as well as expand conventional linear analysis with high-dimensional analysis that can account for the complexities of the microbiome.

We observed both similarities and discrepancies with individual-level studies. Our results are consistent with the study from Yeoh et al., with an increased proportion of Bacteroides and decreased Actinobacteria in COVID-19 patients compared to controls (Yeoh et al., 2021). A small cross-sectional study (n=48) reported a decreased diversity and an increase in the Bacteroidetes phylum associated with increased severity of COVID-19 which is consistent with our findings, as well (Hazan et al., 2022). On the other hand, a study comparing controls with patients of different COVID-19 severity reported an increase in Actinobacteria in Covid-19 patients. They report a decrease in Firmicutes in line with our results, as well as a decrease in microbial diversity as Covid-19 severity increases which we did not find (Wu et al., 2021). The direct comparison (PERMANOVA) identified taxa specifically associated with COVID-19. Yeoh et al. (2021) found COVID-19 patients to have lower quantities of *Eubacterium rectale* and *Bifidobacterium adolescentis*. We identified *Eubacterium rectale* and Bifidobacterium, more specifically the genus *adolescentis*, as the two most beneficial taxa. This perhaps accounts for the lack of such bacterial genera in diseased patients. These genera in particular are also known to influence the immune response, further supporting our finding (Zheng et al., 2020; Baradaran Ghavami et al., 2021).

Considering the harmful taxa as identified by PERMANOVA, *Prevotella copri*, which belongs to the Bacteroidetes phylum, is also known to play a role in the development of rheumatoid arthritis (Alpizar-Rodriguez et al., 2019; Drago, 2019). In a small study comparing COVID-19 patients to controls, a decrease in *Prevotella copri* abundance was noted, however, a positive association with viral load of SARS-CoV-2 in the upper respiratory tract was also identified. Moreover, the same study found an increase in *E.coli* in COVID-19 patients, and a positive correlation with COVID-19 severity (Zhou et al., 2022), which is supported by the PERMANOVA in this study. Though *E.coli* are a commensal member of the gut microbiota, there are multiple well known pathogenic subspecies which may alter different cellular processes (Kaper et al., 2004). In line with other studies, these bacteria suggest a potential, detrimental role in COVID-19 severity, however, neither bacterium was found to be a significant association in the TDA analysis. *Prevotella copri* was identified as a highly enriched taxon and enriched in a similar area of the network graph as the high severity group, but this association did not pass the strict significance threshold.

The unsupervised machine learning with TDA provided additional insights above and beyond those from the PERMANOVA. The bacteria significantly associated with the low group are *Eubacterium rectale, Bifidobacterium longum*, and *bifidum.* These are thought to be anti-inflammatory bacteria and the latter two of which are key strains in many probiotic therapeutics with a host of beneficial effects (Sharma et al., 2021). Strains from these and other probiotics species have previously been shown to have beneficial effects on the incidence and course of other viral respiratory infections, potentially by acting on the gut-lung axis and the innate immune system which makes it a target to reduce inflammation in COVID-19 (Baud et al., 2020; Stavropoulou and Bezirtzoglou, 2020). Indeed, strains of these two Bifidobacteria were used in a probiotic ‘cocktail’ to treat 99 hospitalised COVID-19 patients in a randomised controlled trial (Ivashkin et al., 2021). However, there was no effect of the probiotic on clinical outcomes. The same was found in a smaller case-control study (N=30), although neither the strains nor the dose of probiotics administered was recorded, making that study difficult to interpret (Veterini et al., 2021). While these results may appear to suggest a reduced influence of the microbiome on COVID-19 severity, it is actually in line with the results reported here. Since we take a population-level approach, we consider the ‘baseline’ microbiome of the population. The results thus speak to the potential preventative resilience of the microbiome and our results point towards potential preventative treatments rather than interventional treatments for COVID-19. This underscores the value of population-level approaches as taken in this study for treatment translation. This point is strengthened by the results reported in a recent preprint of an RCT which observed that exposed people who took a daily Lactobacillus-based probiotic were less likely to develop COVID-19 and if they did, to develop it later than controls (Wischmeyer et al., 2022). Together, this suggests that the microbiome might be an accessible and promising target for prophylaxis.

The depleted levels of the Bifidobacteria genus are consistent with other studies (Bozkurt and Bilen, 2021a). Additionally, hospitalised patients that received a *B.animalis* probiotic were reported to have reduced mortality and improved symptomatology, as well as a reduction in IL-6 (Bozkurt and Bilen, 2021b). Such findings are in line with the proposed mechanism of action of Bifidobacteria species on the immune system through affecting dendritic cells of the intestinal mucosa (López et al., 2011). In fact, it has been shown that different Bifidobacteria strains have specific effects on T cell differentiation. For example, there are four *B.bifidum* strains that are Th17 inducing (López et al., 2011). This is particularly interesting as there is now evidence to suggest that Th17, and the Th17 inducer cytokine IL-6, could be a driver of the COVID-19 cytokine storm, although mechanistic insight so far is inconclusive (Paiva et al., 2021). This is supported by previous evidence that suggests the microbiome as a regulator of the Treg/Th17 axis, which is responsible for appropriate protection from pathogens without excessive or autoimmune response (Omenetti and Pizarro, 2015). Together, this evidence is thus highly suggestive of a link between Bifidobacterium levels and COVID-19 severity.

Nevertheless, there are some discrepancies between our findings and the literature. For example, *Ruminococcus torques* had been found to be more abundant in Covid-19 patients whereas our PERMANOVA analysis includes this specific species in the beneficial taxa. Furthermore, the bacteria *Collinsella aerofacis* has been detected in a recent study published in 2020 in Covid-19 hospitalised patients, therefore going against our findings from the PERMANOVA (Zuo et al., 2021). In the TDA co-enrichment analysis, however, this species was associated with the probiotic species associated with the low severity group - *Bifidobacterium bifidum* - and *Eubacterium hallii*, *Ruminoccus obeum*, and *Dorea formicigenerans*. This could suggest an indirect mechanism of action on COVID-19 severity by reducing the abundance of those probiotic species that future studies should explore further. In line with the TDA finding and the Zuo *et al.* study, but not the PERMANOVA finding, the literature suggests a more pro-inflammatory profile of *Collinsella aerofacis*, as it seems to play a role in regulating immunity: it is associated with immunotoxicity in immune checkpoint inhibitor therapy in cancer (Kageyama et al., 2020), with type 2 diabetes (Lambeth et al., 2015), and with rheumatoid arthritis (Chen et al., 2016), although other studies report the opposite (Jeong et al., 2019). In overweight and obese pregnant women, a study found a positive association of the genus *Collinsella* with circulating insulin and a negative association with dietary fibre intake (Gomez-Arango et al., 2018). In our study, the bacterium was associated with normal BMI, as were the taxa it co-enriched with. This discrepancy could be due to different effects of species within a genus. In addition to pointing toward potentially novel mechanisms of action, these findings also support the superior performance of TDA over conventional analysis in picking up subtle and complex relationships, as suggested by Liao et al. (2019).

In addition to these bacterial associations, TDA suggested an association with both individual- and country-level confounders. Our results show associations of the low severity group with the percentage of over 70-year-olds in the population, GDP, and HDI, which were all also associated with each other, and the three anti-inflammatory bacteria associated with low severity. This suggests a confounding effect of these variables on the microbiome-disease severity association. This finding is in line with a previous study looking at between-country variation of the case fatality rate (CFR) which found positive associations between CFR and percentage over 70-year-olds, GDP, and level of democracy (Sorci et al., 2020). Their results suggest that there are highly complex interactions between different facets of healthcare spending, policy, and social or cultural factors that impact the severity of COVID-19. However, they can impact the microbiome as well. The diet of a population changes with population wealth, becoming more diverse and nutrient-rich with rising GDP (Ritchie and Roser, 2017). Diet in turn massively impacts the composition of the microbiome (David et al., 2014). Further, socioeconomic status at the individual level is also associated with changes in microbiome composition (Bowyer et al., 2019). Similarly, the composition of the microbiome changes at an individual level with increasing age (Yatsunenko et al., 2012; Zapata and Quagliarello, 2015; Badal et al., 2020), which could arguably be reflected in the composition of the population microbiome.

Further, both the age distribution of a country, as well as its wealth, impact the number of comorbidities present in that population that could impact COVID-19 severity and the microbiome. This again could be mitigated by the performance of different healthcare systems. There are several different ways in which these three variables and the microbiome-COVID-19 relationship can be related. To disentangle these effects, future research needs to account for further variables such as diet, healthcare spending, healthcare performance, comorbidities, and public health policy.

Additionally, our study supports an association between male gender and low severity, which is contrary to the widely reported increased risk of men for worse COVID-19 outcomes and death (Jin et al., 2020). A large meta-analysis reports that male patients have much higher odds of both requiring intensive care (OR 2.84) and mortality (OR 1.39), which occurs globally (Peckham et al., 2020). This result is likely caused by a gender imbalance in the microbiome dataset: the dataset includes twice as many females as males and the high severity group specifically only contains 5% males. Similarly, low severity was significantly associated with the school-age age category, which is also underrepresented in the high severity group and has a low sample number in general. For variables with few samples which are highly distributed across the network, enrichment is difficult to interpret as permutation has a low probability.

There are several limitations of this study.

The study assesses association and not causality, which would require prospective and interventional studies. Additionally, the range of confounding factors included in our study has been limited due to the availability of data. For example, we do not have extensive metadata on ethnicity, or diet, antibiotics use, and other lifestyle habits of individual samples in our dataset – while this data exists for around 10-15% of subjects in the cMD dataset, it is not broad enough to be used in our analysis. These factors are all known to affect the microbiome (Dethlefsen et al., 2008; Monda et al., 2017; Vaughn et al., 2017; Huang and Shi, 2019), and in the case of ethnicity is also known to affect COVID-19 severity (Berkowitz et al., 2020). Further, by excluding samples with known diseases to avoid confounding in the microbiome dataset, we also limit how representative the included microbiome data is of its population, potentially producing sampling error.

There are similar issues with population-level data: while the observed co-enrichment of the country-level confounders and both the low severity group and significant taxa suggests a considerable confounding or mediating effect, more fine-grained effects were not able to be detected due to a lack of appropriate variables. However, these three variables were chosen based on the existing literature and an *a posteriori* inclusion of further interesting variables to disentangle the observed effects is beyond the scope of this study. Further confounders that we would suggest being included in future studies can be grouped into four archetypes that affect both the microbiome as well as COVID-19 severity. First, confounders reflecting underlying population genetics such as the prevalence of specific COVID-19 prognostic risk alleles (Fricke-Galindo and Falfán-Valencia, 2021) that could have systematic effects on microbiome composition (Goodrich et al., 2014). Second, confounders reflecting individual lifestyle, such as smoking, which are known to affect both COVID-19 severity (Gao et al., 2021) and the microbiome (Savin et al., 2018). Third, confounders reflecting the healthcare system as a proxy measure of a country’s ability to treat COVID-19 such as the rate of ICU beds or ECMO capabilities, but also as a proxy measure of the general health of a country as reflected by healthcare expenses per person. Finally, population health measures and policy during the pandemic should be included as they have an effect both on COVID-19 prevalence and severity (Huang et al., 2021; Arnold et al., 2022), as well as on the microbiome due to lifestyle changes induced by lockdowns and increased hygiene (Burchill et al., 2021; Finlay et al., 2021).

Further, due to the scarcity of samples and confounding variables, multiple countries ought to be excluded. The study may have benefitted from the inclusion of more countries to observe a full geographical variation. On the same note, we have noted an uneven distribution of microbiome samples across the countries, with an overall range of gut microbiome samples from 24 in Estonia to 900 in Israel, although using the weighted mean as a threshold for the low and high groups of disease severity allowed the groups to be even. Of course, a key assumption of this study is that the observed samples are representative of their populations. Even though a larger sample size and more evenness between countries would increase the likelihood of that assumption holding true.

Another limitation lies in the metric chosen to assess COVID-19 severity. The hospitalisation rate can be influenced by factors such as the healthcare system itself and the pressure on that system at any moment during the pandemic. Other potentially influential factors can revolve around public policy and in particular public health measures such as social distancing and lockdowns, as infection rates and thereby hospitalisations could have been affected. In light of these limitations, future studies should therefore aim to investigate the causal role of the gut microbiome in COVID-19 severity and further examine the long-term effects of the gut microbiome.

The findings of this study, together with similar findings in the literature, have translational potential. Considering the strong associations between the gut microbiome and the severity of COVID-19, future studies should look further into specific mechanisms with a particular emphasis on the possible beneficial role of prebiotics and probiotics and targeted dietary interventions.

Additionally, as previous studies have demonstrated antiviral activity of specific probiotic strains in several respiratory viruses, including other coronaviruses and COVID-19, special attention should be paid to more targeted approaches (Baud et al., 2020; Bozkurt and Bilen, 2021b). The need for strain-level studies is supported by the finding that different strains of the same species can have differential effects on T cell differentiation López et al., 2011. This includes individual- and population-level studies at the strain level, as well as targeted case-control studies and RCTs. However, the largest effect of probiotics may be exerted when delivered as a public health intervention. As this study suggests, a more anti-inflammatory microbiome profile may be a contributor to fewer hospitalisations from COVID-19 - with effects not only for the individual but for the whole country. Less pressure on the healthcare system could mean potentially fewer excess deaths due to cancelled routine appointments and planned surgeries as has been observed especially for cancer patients during the pandemic (Maringe et al., 2020; Riera et al., 2021). The microbiome is a particularly accessible treatment target for population-level interventions, as it can be modulated through oral supplements or diet. Similar to how foods, such as flour and salt, and other products, such as toothpaste, have been fortified to successfully prevent birth defects (Brown et al., 2011), iodine deficiency (Andersson et al., 2010), and caries (Twetman et al., 2003), respectively, such everyday products could be fortified to improve the microbiome.

### 4.1 Conclusion

The findings of this study suggest an association between the microbiome of country populations at baseline and the severity of COVID-19 experienced during the pandemic, as measured by hospitalisations. Specifically, anti-inflammatory bacterial taxa including *Bifidobacteria* and *Eubacterium rectale* were identified as protective. We also find evidence that characteristics of countries, such as the proportion of over 70-year-olds, as well as GDP and Human Development Index, influence this association between the microbiome and COVID-19 severity. Future studies should aim to disentangle the direct and indirect effects of wealth, policy, and population characteristics on the microbiome and on hospitalisations. These findings were made possible by a unique study design of indirect association using open-access population-level data. As our findings both support and expand upon individual-level findings, this study highlights the unique capabilities of such population-level studies for understanding diseases and finding novel treatment avenues, especially when considering the microbiome which is a particularly accessible target. Finally, the use of an unsupervised machine learning tool, Topological Data Analysis, strengthened and expanded findings from conventional microbiome analysis, particularly through its ability to account for nonlinear relationships and the effect of confounders. This validates TDA as a valuable tool for microbiome analysis, particularly for microbiome-disease associations.

## Data availability statement

Publicly available datasets were analyzed in this study. This data can be found here: https://bioconductor.org/packages/release/data/experiment/html/curatedMetagenomicData.html and https://github.com/owid/covid-19-data.

## Author contributions

EL and GG: Conceptualization, methodology, formal analysis, writing – original draft, writing – review & editing, visualization, project administration. SB: Writing – review & editing. NS: Conceptualization, methodology, formal analysis, writing – review & editing, visualization, supervision, project administration, funding acquisition. All authors contributed to the article and approved the submitted version.

## Funding

This research was supported and funded by a grant from the Reta Lila Weston Trust. EL is supported with a studentship by the EPSRC (training grant number EP/S021612/1). SB is funded by the Wellcome Trust (Grant Reference 566701). EL and NS are supported by the National Institute for Health Research University College London Hospitals Biomedical Research Centre.

## Conflict of interest

Author NS is CEO and founder of BioCorteX Ltd. Author SB owns equity in Owkin Inc.

The remaining authors declare that the research was conducted in the absence of any commercial or financial relationships that could be construed as a potential conflict of interest.

## Publisher’s note

All claims expressed in this article are solely those of the authors and do not necessarily represent those of their affiliated organizations, or those of the publisher, the editors and the reviewers. Any product that may be evaluated in this article, or claim that may be made by its manufacturer, is not guaranteed or endorsed by the publisher.
